# Dental Exhibition at the World's Fair

**Published:** 1852-07

**Authors:** 


### ARTICLE VI.

### *Dental Exhibition at the World's Fair.*

The English metropolis has again resumed its usual business-like appearance, and its inhabitants have betaken themselves to their accustomed avocations; they are no longer jostled and hustled by the *starving*, fat-faced merry-looking thousands of agriculturists from the provinces, or questioned by foreigners with strange physiognomies, and still stranger costumes; in fact, Mr. John Bull has London to himself again, and merely looks upon the present state of the exhibition as the remains of its former greatness, and as no inapt type of what we are informed the New Zealand traveler will one day behold, when standing upon an arch of London bridge, he surveys the ruins of St. Paul's. It is not, however, to the New Zealand traveler, or the present state of the crystal palace, or the thousands of complaints of the different exhibitors in the different sections to which we wish to direct the notice of our readers. The various branches of the arts and sciences, have doubtless their own periodicals, by means of which, their wrongs can be brought before the public. Our duty is to direct the attention of our profession to the jurors appointed as judges in section X, in the great exhibition, a portion of which may be fairly said, represents our profession. The gentlemen appointed to preside over this portion of the exhibitors, were not, from the nature of their calling and education upon such matters, calculated to estimate rightly the various dental mechanism, dental appliances, porcelain work, &c., submitted to their inspection; and even had they sought an explanation from the exhibitors themselves, (which they did not,) we have no hesitation in saying, they could not, by any possibility, possess the requisite competency to arrive at a just conclusion so as to form a correct judgment as to the scientific construction and applicability of the articles submitted. We presume, therefore, the jurors discovered their own incompetency in this department of the section, and therefore contented themselves with the mere inspection and the awarding of prizes for the best collection of physician's, surgeon's, aurist's and occulist's instruments and mechanical arrangements, which, to do the instrument makers justice, had taken especial pains to display to a *trading* and attractive advantage, by exhibiting their wares in immense show cases. Amongst the whole of this *collection*, for which prizes were awarded, we may safely assert, there was not one (excepting the dental instrument makers) which could be fairly termed modern instruments, used by dental practitioners. Yet these wise-acre professionals, who had so unceremoniously accepted this important office, as unceremoniously *dismisses* the whole batch of dental exhibitors, whose contributions, as we before observed, they could not, by education or practice, properly appreciate, as a poor law-relieving officer dispatches the applicants from his office without a thought or consideration. Surely the dental practitioners of Europe will well know how to appreciate the feelings of these *remarkable* jurors who hold their profession in such high consideration.

With these remarks, we will proceed to notice the various specimens of dental mechanism exhibited at the great exhibition in England in 1851. It would appear from the official catalogue, that there were no more than from eighteen to twenty practical dentists in England, Scotland and Ireland, who sent specimens of their mechanical skill to the exhibition, amongst which we noticed the following:

329.—Messrs. Sinclair & Hockley—A number of specimens of different kinds of artificial work, which were neither well finished or original in design.

441.—Mr. Waite—Specimens of gold blocks set with precious stones, on grinding surface ; also, a bottle containing a strong acid, in which were deposited gold plates for the purpose of illustrating, that if a similar standard of metal be worn in the mouth, no chemical action takes place from the action of the saliva.

558.—Mr. Nolan—Exhibited an excellent specimen of mineral teeth, set in bone, highly finished, and artistically made; also, an assortment of old fashioned and roughly made swivels.

574.—Mr. Ghrimes—A number of specimens of natural and mineral teeth, mounted in bone sockets. The workmanship and finish of these specimens, deserved especial notice from the profession.

575.—Mr. Horne—A case containing several pieces of dental mechanism and regulating plates, &c. These we only notice for the inferior workmanship and bad finish.

576.—Mr. Laurie—A beautiful carved set of artificial teeth from the tusk of the hippopotamus. This was the most highly finished and artistically carved specimen, of this description, in the whole exhibition.

579.—Mr. Perkins—Several well executed pieces of bone work.

583.—Dr. Reid—Exhibited an ingenious compress for arresting dental hemorrhage, showing its applicability on a cast of the head, &c.

584.—Mr. Rausom—A set of Ash's gum teeth, badly fitted to gold plates.

601.—Mr. Miles—A number of pieces of artificial work, one of which, in bone, representing an upper set, was well executed and original in conception. This gentleman was the only practitioner who exhibited a full and particular list of the *prices* of his handicraft.

602.—Mr. Finsi—A set of teeth of inferior workmanship—a universal dental drill, consisting of a beveled wheel, fixed to a handle, acting on another attached to the shaft. It is a clumsy and unwieldy machine, which, if we remember correctly, is used by gun makers, &c.

636.—A *patented* instrument exhibited by Mr. Goddard for extracting teeth perpendicularly; invented and *patented* by E. Browne, of New Bedford, Mass., America.

643.—Mr. Wood—A series of casts, illustrating the application of an old plan newly revived, for correcting and preventing irregularities of the permanent teeth, by means of a plate and screws; also, several rough and unfinished pieces of artificial work.

717.—Mr. Watt—A number of pieces of artificial work, excellently carved and finished, from the tusk of the hippopotamus.

718.—Mr. Dinsdale—A very inferior set of block porcelain teeth. Two casts of the mouth, illustrating the effect of artificial teeth in restoring the symmetry of the face.

719.—Mr. Rose—Teeth mounted upon gold; beautiful for its execution and finish.

720.—Mr. Trumen—Various kinds of teeth, mounted upon a basis of gutta percha, colored, and intended to represent the human gums.

721.—Mr. Harrington—An apparatus for moulding tortoise-shell for the beds of artificial teeth, a clever and ingenious apparatus for the purpose, but from the unmanageableness of the material, and its contracting properties, the whole process has been discarded by the profession as worse than useless.

*Manufacturers of Porcelain Teeth.*

578.—Messrs. Ash & Sons—A large assortment of artificial teeth and gum blocks, with gold tubes. The teeth, for form, color, and natural appearance, are unquestionably amongst the best in the exhibition. The block pieces are too flat at the edges intended to represent the gum, giving them an unnatural and formal appearance.

633.—Mr. Harnett—A large case, containing various instruments and materials used by dentists, with inferior specimens of block teeth.

*Dental Instrument Makers.*

640.—Mr. Weedon—Various well finished dental instruments of modern patterns.

647.—Mr. Everard—Enamel scrapers, showing their hardness of temper on glass; a number of highly finished forceps, with straight handles and beaks; also, the various stages of the instruments during the progress of manufacture.

678.—Mr. Jack—A number of well finished forceps, with straight handles and beaks.

*France.*

The dentists of this country were represented by only one exhibitor—a *Mons. Simon*—a rather ominous name for the respectability and progress of the profession, if we are to judge the present state of the mechanical art from the specimens exhibited, consisting of several roughly, inartistically made wire pieces, blocks of sea-horse, fashioned to represent teeth, a few caricatures of attempts at plate-making of the worst description.

*Canada.*

193.—Mr. Dickinson—A very excellent specimen of a pivoted American mineral, with section of tooth, showing the pin in root.

19.—Mr. Rahee—A well executed piece of dental mechanism, consisting of a set of gold plates, with tube teeth, set in sockets, retained in the mouth with spiral springs.

*United States.*

9.—Mr. Hunter—A piece for the lower jaw in gold, badly mounted, with porcelain teeth and worse finished.

47.—Mr. Wardle—Five plates and one full set.

61.—Mr. Reynolds—Two sets and two pieces admirably mounted and highly finished; not excelled in the exhibition.

63.—Mr. Buckingham—Illustrations of the various processes of making a set of teeth, with a very bad sample of zinc casting, several inclined planes for regulating teeth roughly made, in metal.

350.—Mr. Barlow—A well executed piece of mineral work with socket rim.

518.—Mr. Hitchcock—A well made set, in block, mounted on gold, with sockets.

558. ——— Philadelphia—Samples of teeth filled with gold, with sections showing the compactness of the filling. These are amongst the most beautiful specimens of pluggings we have ever witnessed, and the exhibitor, whoever he may be, need not have withheld his name.

*Manufacturers of Porcelain Teeth.*

33.—Messrs. Jones, White and McCurdy—A fine collection of porcelain teeth, with a great variety of shades, &c.; specimens of Hill's stopping, a most excellent substitute for the mercurial paste.

220.—Mr. Alcock—A large assortment of teeth of uniform quality, some of which, we were glad to observe, contained tubes similar to those made in England. If American manufactures were to turn their attention to this description of teeth, with their improved shades and *natural* shapes, for the English markets, we doubt not they would have an extensive demand, if sold at a more moderate rate.

419.—Mr. Stockton—Thousands of excellent teeth of every variety of shape and color. The molars in this collection appeared much too dark as a substitute for the natural organs.

*Instrument Makers.*

120.—Mr. Chevalier—a most beautiful collection of highly finished instruments, chiefly used by American dental practitioners, amongst which we noticed Dr. Hullihen's screw stump forceps. This exhibitor deserves the thanks of the profession for the excellent selection he made, and the superior taste displayed in their finish and manufacture. R.

